# Digital transformation in community pharmacy management: Pharmacists' readiness for cloud computing systems, implementation concerns, and adoption support

**DOI:** 10.1016/j.rcsop.2026.100845

**Published:** 2026-09-15

**Authors:** Mohanad Odeh, Dana M. Odeh, Wafa Hourani, Ahmad Shadfan, Feras Jirjees

**Affiliations:** a Department of Clinical Pharmacy and Pharmacy practice, Faculty of Pharmaceutical Sciences, The Hashemite University, Zarqa, Jordan; b Department of Pharmacy, Pharmacy School, Jadara University, Jordan; c Faculty of Pharmacy, Philadelphia University, Amman, Jordan; d Medicine School, The Hashemite University, Zarqa. Jordan; e Department of Clinical Pharmacy and Pharmacy practice, Faculty of Pharmaceutical Sciences, The Hashemite University, Zarqa, Jordan

**Keywords:** Pharmacy management systems, Cloud computing [MeSH], Community pharmacy services [MeSH], Pharmacists [MeSH], Pharmacy [MeSH], Medical informatics applications [MeSH], Diffusion of innovation [MeSH]

## Abstract

**Background:**

Cloud computing is increasingly positioned as a key enabler of digital transformation in pharmacy practice, offering potential benefits for information access, workflow flexibility, data storage, backup, and service continuity. However, pharmacists' readiness to adopt cloud-enabled pharmacy systems remains insufficiently examined, particularly in community pharmacy settings.

**Objective:**

This study assessed pharmacists' readiness for cloud computing adoption as part of digital transformation in community pharmacy management, focusing on perceived usefulness, digital-service orientation, service quality, cost and value, security and privacy, infrastructure readiness, backup and recovery, and demographic predictors of adoption support.

**Methods:**

An analytical cross-sectional study was conducted using a validated electronic questionnaire. Eligible participants were licensed pharmacists with current or previous community-pharmacy experience. Descriptive statistics were used to summarize response patterns, chi-square tests examined bivariate associations, and Cramér's V was used to quantify the strength of statistically significant associations. Multivariable logistic regression identified independent predictors of readiness and support for cloud-service adoption.

**Results:**

Pharmacists showed strong receptiveness toward cloud-enabled pharmacy services. Most respondents agreed or strongly agreed that cloud computing could be useful for the pharmacy sector (89.1%), support wider electronic pharmaceutical services (93.7%), improve service quality (89.6%), and improve service flexibility (89.1%). However, uncertainty remained regarding cost reduction, return on investment, start-up cost, internet reliability, offline continuity, and privacy/security risks. In multivariable regression analysis, female pharmacists had higher odds of supporting cloud services than male pharmacists (OR = 7.024; 95% CI: 1.60–30.72; *p* = 0.010), and pharmacists aged ≤35 years had higher odds than older pharmacists (OR = 5.778; 95% CI: 2.31–14.39; *p* < 0.001). The model demonstrated good discrimination (AUC = 0.820; 95% CI: 0.683–0.956).

**Conclusion:**

Pharmacists demonstrated high perceived readiness for cloud computing adoption, but implementation depends on resolving cost, connectivity, offline-continuity, and privacy/security concerns. A data-informed and Diffusion of Innovations-informed readiness framework is proposed to guide future validation and implementation planning.

## 1 Introduction

Pharmacy practice services are increasingly exposed to a staged digital transition, moving from paper-based records and local on-premises computer software^1^ toward integrated, cloud-enabled platforms and, more recently, AI-supported management systems.^2^, ^3^ Pharmacy management systems (PMS) have been described as established technologies supporting hospital and community pharmacy functions, including medicine labelling, ordering, stock control, integration, reporting, and service-support applications.^4^ Ideally, this digital transformation should strengthen medication management across hospitals, primary-care units, and community or retail pharmacies, as cloud computing enables shared and scalable access to applications, storage, and services, while AI has emerging applications in medication management, decision support, and patient care.^5^, ^6^

In hospital and primary-care settings, digital medication technologies—including electronic prescribing, computerized provider order entry, electronic medication-management systems, and medication-related clinical decision support—have been extensively evaluated. Systematic reviews and evidence syntheses suggest that these interventions can reduce prescribing and medication-error rates, although their impact depends on system design, workflow integration, usability, implementation quality, and professional oversight.^7^, ^8^, ^9^, ^10^, ^11^, ^12^, ^13^, ^14^, ^15^, ^16^, ^17^

In contrast, community-pharmacy evidence remains comparatively limited.^18^ Existing studies have mainly examined discrete digital medication services rather than comprehensive cloud-based PMS, including medication safety^19^ or early mobile connected pharmacy models,^20^ shared electronic health records such as My Health Record, real-time prescription monitoring, and electronic prescriptions,^21^ drug monitoring programs in the United States,^22^ electronic prescribing in community pharmacies,^23^, ^24^ virtual care in community pharmacy,^25^ Telepharmacy services^26^, ^27^ and to some extent some Telehealth initiatives as teleconsultation, telemonitoring, telecollaboration, and telesupport.^28^ These studies have expanded understanding of digital medication services in community settings. However, they focus mainly on discrete digital medication services rather than pharmacists' readiness to adopt cloud-based and emerging AI-supported PMS.

Although fewer studies have addressed comprehensive PMS directly, software-development studies and digital-health initiatives increasingly describe or promote PMS platforms for community pharmacies, including systems for dispensing support, inventory monitoring, expiry-date tracking, sales reporting, prescription handling, analytics, patient follow-up, and related operations.^2^, ^3^, ^29^, ^30^, ^31^, ^32^ Such literature largely emphasizes system functionality rather than pharmacists' readiness, including perceived usefulness, trust in AI outputs, privacy concerns, cost acceptability, workflow compatibility, governance expectations, and practical acceptability.

Therefore, assessing community pharmacists' readiness to transition from traditional on-premises software toward cloud-based and emerging AI-supported PMS is necessary before large-scale implementation, particularly in settings where commercial digital solutions may be advancing faster than independent evidence on adoption, governance, safety, and practical acceptability.

## 2 Theoretical and conceptual orientation

To clarify the technological scope of the present study, Table 1 summarizes the staged transition from traditional pharmacy work toward cloud-enabled and AI-supported cloud pharmacy systems. The table is not intended to present a strict historical sequence; rather, it provides a conceptual distinction between paper-based practice, local on-premises software, electronic pharmacy services, integrated digital pharmacy workflows, cloud-enabled systems, and AI-supported cloud platforms.^2^, ^3^, ^4^, ^33^ To establish strict terminological consistency throughout this study, explicit definitions are maintained across distinct layers of digital transformation: cloud computing refers to the underlying IT infrastructure model providing remote data storage and processing; cloud-based PMS denote the specific core application software hosted on cloud infrastructure for daily dispensing and inventory operations; and cloud-enabled pharmaceutical services represent the broader clinical, remote, and collaborative patient-care functions facilitated by remote connectivity.

**Table 1:** Conceptual stages of digital transformation in pharmacy practice.

Stages⁎	Core system	Community pharmacy example	Main value
Traditional pharmacy	Paper/manual records	Paper prescription, manual stock count	Direct human service
On-premises software	Local pharmacy computer/server	Local POS, billing, inventory, patient profile	Basic digital organization
Electronic pharmacy	*E*-prescriptions and EMRs	Electronic prescription received from clinic	Less paper, faster workflow
Digital pharmacy	Integrated pharmacy workflow	Dispensing + insurance + barcode + inventory linked	Safer, more efficient practice
Cloud-enabled pharmacy	Real-time shared ecosystem	Branches share inventory, refill data, patient records	Connectivity and scalability
AI cloud-enabled pharmacy	Cloud + analytics + AI models	Predicts stock needs, flags risky prescriptions, supports adherence follow-up	Predictive, personalized care

PMS, pharmacy management system; EMR, electronic medical record; POS, point-of-sale system.

⁎ The stages illustrate the broader technological trajectory of pharmacy management; while cloud-enabled systems constitute the direct empirical focus of this study, subsequent AI-supported integration stages are presented strictly as contextual background and future transformation components rather than measured variables.

In this study, cloud services refer to internet-enabled external services that allow pharmacy-related data, applications, storage, processing, and service functions to be accessed through remote network-hosted servers rather than being restricted to local computers or on-premises hardware. This definition is consistent with the National Institute of Standards and Technology definition of cloud computing as on-demand network access to shared configurable computing resources, including networks, servers, storage, applications, and services.^4^, ^34^

The study was guided by a readiness-domain approach and Rogers' Diffusion of Innovations theory.^35^ The readiness-domain structure organized pharmacists' evaluations across core implementation dimensions, including perceived usefulness, digital-service orientation, service quality and flexibility, cost and value, security and privacy, infrastructure readiness, and backup and recovery. Rather than serving as directly measured psychometric constructs, Rogers' DOI dimensions were applied post-hoc as an interpretive lens, recognizing that cloud computing adoption in pharmacy practice represents a complex professional and workflow-related innovation rather than a routine software preference. Within this theoretical mapping, perceived usefulness and service quality/flexibility align with *relative advantage*; security, privacy, and data backup/recovery map to *compatibility*; and cost evaluations correspond to *complexity*. Consequently, these DOI principles—alongside trialability, observability, and confirmation—contextualized pharmacists' behavioral readiness and guided the architecture of the proposed adoption-readiness framework.

This study aimed to assess pharmacists' readiness for cloud computing adoption as part of digital transformation in community pharmacy management, focusing on perceived usefulness, digital-service orientation, service quality and flexibility, cost and value, security and privacy, infrastructure readiness, backup and recovery, and overall support for cloud-enabled pharmaceutical services. The study also examined demographic and professional predictors of cloud-adoption readiness (operationalized as supportive versus uncertain/non-supportive states**)** as well as to develop a proposed data-informed and DOI-informed framework to summarize adoption readiness and decision-making in community pharmacy. By identifying readiness patterns and implementation concerns among pharmacists, this study provides exploratory evidence to inform future implementation strategies, intervention design, and theory-driven research on cloud-enabled community pharmacy services.

## 3 Methodology

### 3.1 Study design and reporting approach

This analytical cross-sectional study used a validated electronic questionnaire to assess community pharmacists' perceptions and readiness toward cloud computing adoption in pharmacy practice. The study focused on perceived usefulness, digital-service orientation, service quality, cost and value, security and privacy, infrastructure readiness, backup and recovery, and overall support for cloud-enabled pharmaceutical services. Reporting was guided, where applicable, by the STROBE checklist for cross-sectional studies and the CHERRIES checklist for internet-based surveys.^36^, ^37^ The study background acknowledges the broader digital evolution toward AI-supported platforms (Table 1), whereas the empirical scope of this investigation focuses exclusively on pharmacists' readiness for cloud computing adoption.

### 3.2 Study setting and eligibility criteria

The study was conducted in Jordan between February and September 2025. Eligible participants were licensed pharmacists in Jordan with current or previous exposure to community pharmacy practice, including those working in individual or chain community pharmacies, as well as pharmacists currently employed in pharmacy-related sectors but with relevant community-pharmacy experience. It is noteworthy that within the Jordanian regulatory context, every community pharmacy must operate under one designated ‘licensed responsible pharmacist’ (pharmacist-in-charge). In both individual pharmacies (where the pharmacist is frequently the owner) and chain pharmacies (where the licensed pharmacist serves as the branch manager), these professionals hold direct managerial responsibility and are expected to plan, evaluate, and contribute to operational decisions regarding pharmacy management systems. Additionally, other community pharmacy setting representing licensed responsible pharmacists within the emerging transitional phase between independent and chain pharmacy models, as recognized in contemporary Jordanian pharmacy practice literature.^38^ Participants were required to be able to complete the electronic questionnaire. Community pharmacists were selected because they represent key end-users of pharmacy management systems and the adoption of cloud-based pharmacy services. Pharmacists with no relevant exposure to community pharmacy practice were not eligible.

### 3.3 Participant recruitment and selection

Participants were recruited using non-probability convenience sampling through Facebook pages used by Jordanian pharmacists, active WhatsApp groups including community pharmacists, and a community-pharmacist database available at the Hashemite University. Multiple electronic recruitment routes were used to extend outreach across different professional networks**.** Participation was voluntary, and online informed consent was obtained before survey completion. Google Forms settings were configured to allow only one response per participant, reducing the risk of duplicate submissions.

### 3.4 Variables

The primary outcome for multivariable analysis was pharmacists' readiness for cloud adoption, operationalized as a binary readiness state derived from the core cloud-service support item. Conceptually, readiness represents the overarching evaluative state of the pharmacist, while support for adoption serves as its behavioral indicator. Respondents whose questionnaire feedback indicated more positive overall evaluations on this core item were categorized as ready (supportive), whereas those with negative or neutral views were classified as not ready (uncertain or non-supportive). To maintain strict terminological consistency with the study's primary objective and title, “readiness for cloud adoption” is used as the overarching construct throughout the manuscript, with “supportive versus uncertain/non-supportive” defining the specific operational states of that readiness. Independent variables included gender, age group, education level, place of work, and years of professional experience, all analyzed using predefined categories.

### 3.5 Data sources and measurement

Data were collected using a structured electronic questionnaire developed by the research team in Arabic. To support linguistic clarity and conceptual equivalence, the Arabic questionnaire was translated into English and reviewed through a back-translation and reconciliation process. Discrepancies in wording, meaning, or technical terminology were resolved through research-team discussion before finalization.

The final questionnaire included demographic and professional characteristics followed by Likert-type items across five domains: perceived usefulness and digital-service orientation; service quality and flexibility; cost and value evaluation; security and privacy; and access, backup, and recovery. Responses were measured on a five-point Likert-type scale: strongly agree, agree, not sure, disagree, and strongly disagree.

For inferential analyses, Likert-type responses were grouped where appropriate to compare supportive responses with uncertain or non-supportive responses across demographic and professional groups. For scale-level interpretation, negatively worded items were reverse coded so that higher scores consistently reflected greater readiness or lower resistance toward cloud computing adoption. For descriptive reporting, items were presented in their original wording to preserve interpretability.

### 3.6 Content validity and reliability

Content validity was assessed by a multidisciplinary expert panel of eight members. Panelists were selected based on specific criteria, including a minimum of five years of professional or academic experience in pharmacy practice, health informatics, or cloud computing. The panel comprised two academic experts in clinical pharmacy and health informatics, five experienced community pharmacists—including a designated member of the Community Pharmacists Committee at the Jordanian Pharmacists Association—and one information technology and cloud-services specialist. Through structured expert review and panel discussion, relevant factors were identified, refined, and prioritized.

Item-level content validity was evaluated using Lawshe's Content Validity Ratio.^39^, ^40^ Only items achieving a CVR above 0.741, corresponding to *p* < 0.05 for the expert-panel size, were retained. Out of the initial pool of 40 items, 5 items were excluded as the CVR < 0.741, while 15 items addressing external or environmental constructs (such as legal, regulatory, and system-level policies) were removed from the final readiness questionnaire and reserved for a separate study focused on pharmacists' legal and regulatory awareness of cloud computing, resulting in a final questionnaire of 20 items, in addition to 5 demographics related questions.

Internal consistency was assessed using Cronbach's alpha for the multi-item questionnaire domains. The final item distribution and internal consistency reliability for each domain were as follows: (1) Perceived usefulness and digital-service orientation (two items, Cronbach's α = 0.85); (2) Service quality and flexibility (two items, Cronbach's α = 0.88); (3) Cost and value evaluation (six items, Cronbach's α = 0.84); (4) Security and privacy (six items, Cronbach's α = 0.76); and (5) Access, backup, and recovery (four items, Cronbach's α = 0.79). All retained domains demonstrated Cronbach's alpha coefficients above 0.70, indicating satisfactory internal reliability.

### 3.7 Bias and response-quality control

Several procedures were used to minimize potential sources of bias and improve response quality. The questionnaire was anonymous, which may have reduced social desirability bias, and multiple recruitment routes were used to improve access to pharmacists across different community-pharmacy networks. Google Forms settings were configured to allow only one response per participant, reducing the risk of duplicate submissions. After data collection, responses were screened for completeness, duplication, and consistency between positively worded and reverse-coded items. Responses were excluded if they were incomplete, duplicated, or showed poor internal consistency across coded and reverse-coded items. Based on this quality-control procedure, 23 responses were removed before analysis, leaving 394 valid responses. To reduce confounding, bivariate analyses were first conducted to examine crude associations between demographic/professional variables and questionnaire responses, followed by multivariable binary logistic regression including all prespecified demographic and professional predictors in the same model.

### 3.8 Study sample size

The required sample size was estimated using the Survey Monkey sample-size calculator.41., 42 The calculation was based on an estimated population of 3700 registered community pharmacies in Jordan, according to the Jordanian Pharmacists Association.^43^ Because each Jordanian community pharmacy legally requires one designated responsible pharmacist, the 3700 pharmacy figure serves as a direct 1:1 proxy for the target pharmacist population in the present study. A 95% confidence level and 5% margin of error were applied, with a conservative response distribution assumption of 50% to maximize variability. Based on these parameters, the minimum required sample size was 349 participants. The final analytical sample included 394 valid responses, exceeding the minimum required sample size and supporting the adequacy of the sample for the planned cross-sectional analysis. The total number of unique eligible pharmacists reached through recruitment channels could not be determined; consequently, an overall survey response rate could not be calculated.

### 3.9 Statistical methods

Statistical analyses were conducted using IBM SPSS Statistics for Windows, version 27.0 (IBM Corp., Armonk, NY, USA). Categorical variables were summarized as frequencies and percentages. Descriptive analyses were used to present participant characteristics and response distributions across questionnaire items. Bivariate associations between demographic/professional variables and questionnaire items were examined using categorical chi-squared association test. The demographic and professional variables examined were gender, age group, education level, place of work, and years of experience. The strength of statistically significant associations was quantified using Cramér's V and interpreted as weak when >0.05, moderate when >0.10, strong when >0.15, and very strong when >0.25.^44^

A multivariable binary logistic regression model was conducted to identify independent predictors of readiness for adopting cloud services while adjusting for potential confounding by demographic and professional characteristics. The dependent variable was readiness for adopting cloud services, coded as a binary outcome derived from the total composite score of all five domains (supportive versus uncertain/non-supportive). While this binary transformation facilitates clear, actionable interpretation and robust odds ratio estimations, it inherently involves a methodological trade-off, resulting in some loss of granular variance across intermediate Likert categories. However, fine-grained item-level response distributions across all five adoption-readiness domains are fully preserved and detailed in results section (Table 3) to maintain complete descriptive transparency entered simultaneously into the model were gender, age group, education level, place of work, and years of experience, using indicator contrasts for categorical variables. Results were reported as odds ratios with 95% confidence intervals and *p*-values. Model fit and performance were assessed using the Omnibus test of model coefficients, pseudo-R^2^ measures, the Hosmer–Lemeshow goodness-of-fit test, and the area under the receiver operating characteristic (ROC) curve. The logistic regression included all 394 valid cases, with no missing cases in the model. Statistical significance was set at *p* < 0.05.

### 3.10 Framework development

Following the quantitative analysis, a proposed framework for cloud computing adoption readiness in community pharmacy was developed using a data-informed and theory-informed approach. First, the main enablers and implementation concerns were identified from the descriptive response patterns across the questionnaire domains, including perceived usefulness, service quality and flexibility, cost and value evaluation, security and privacy, and access, backup, and recovery. Second, statistically relevant demographic predictors from the multivariable logistic regression were considered to contextualize subgroup differences in adoption readiness. Third, these empirical findings were organized alongside Diffusion of Innovations theory to illustrate a conceptual adoption pathway from awareness and persuasion toward decision-making, implementation, confirmation, and long-term use. The framework was developed as an interpretive synthesis of the empirical findings and DOI-informed adoption logic rather than as a separately validated prediction model; therefore, it should be considered a proposed framework requiring future psychometric, predictive, and implementation-based validation in community pharmacy practice settings.

### 3.11 Ethical considerations

The study received ethical approval from the Institutional Review Board of the Hashemite University, Jordan (protocol no. 1/2/2019/2020). The protocol was approved prior to the COVID-19 pandemic, project execution and data collection were subsequently rescheduled, with institutional approval remaining fully valid for the active data-collection period between February and September 2025. Online informed consent was obtained from all respondents before participation.

The questionnaire was administered anonymously and did not collect names, national identification numbers, workplace identifiers, telephone numbers, or other direct personal identifiers. Data were stored and analyzed in de-identified form, and all findings are reported in aggregate to prevent identification of individual respondents or workplaces.

## 4 Results

### 4.1 Participants demographics and characters

In the study, total considered responses were 394 responses. The majority of respondents were female (72.1%), with males comprising 27.9%. Participants aged ≤35 years comprised 76.6% of the sample, while those aged >35 years represented 23.4%. Regarding education, 76.9% held a university bachelor's degree, 14.7% had a Pharm.D education, and 8.4% were postgraduates. In terms of experience, 36.3% had 1 to 3 years of experience, followed by 15.5% with less than one year, and 19.5% with over 10 years. Most respondents worked in individual pharmacies (77.4%), while 12.9% worked in chain pharmacies, and 9.6% in non-community pharmacy settings. See Table 2.

**Table 2:** Demographic for participants (*n* = 394).

Variable	Category	Frequency	Percent
Gender	Female	284	72.1
	Male	110	27.9
Age Group	≤35 years	302	76.6
	>35 years	92	23.4
Education level	Pharm.D	58	14.7
	Postgrad	33	8.4
	University Bch	303	76.9
Years of experience	less than one year	61	15.5
	1 to 3 years	143	36.3
	4 to 7 years	73	18.5
	8 to 10 years	40	10.2
	More than 10 years	77	19.5
Place of work	Individual pharmacy	305	77.4
	Chain pharmacy	51	12.9
	Other community pharmacy setting	38	9.6

**Table 3:** Questionnaire item-level response distributions and significant demographic associations across cloud computing adoption-readiness domains.

Domain/Statement^a^	Strongly agree	Agree	Not sure	Disagree	Strongly disagree	Significant demographic association (Bivariate Analysis)
Perceived usefulness and digital-service orientation						
Cloud computing services can be useful for the pharmacy sector.	87 (22.1)	264 (67.0)	39 (9.9)	4 (1.0)	0 (0.0)	None
Pharmaceutical services should adopt more electronic services in the digital era.	110 (27.9)	259 (65.8)	18 (4.6)	7 (1.8)	0 (0.0)	None

Service quality and flexibility						
Adopting cloud technology instead of traditional systems would improve the quality of pharmaceutical services.	115 (29.2)	238 (60.4)	39 (9.9)	2 (0.5)	0 (0.0)	Age: ≤35 vs >35 agreeing/strongly agreeing, 92.1% vs 81.5%; *p* = 0.004; Cramér's V = 0.14
Adopting cloud technology instead of traditional systems would improve the flexibility of pharmaceutical services.	122 (31.0)	229 (58.1)	38 (9.6)	5 (1.3)	0 (0.0)	None

Cost and value evaluation						
Adopting cloud technology would reduce the cost of pharmaceutical services.	13 (3.3)	113 (28.7)	142 (36.0)	100 (25.4)	26 (6.6)	Gender: females vs males agreeing/strongly agreeing, 71.5% vs 59.1%; *p* = 0.018; Cramér's V = 0.12
Adopting cloud technology would increase the cost of pharmaceutical services.⁎	1 (0.3)	75 (19.0)	149 (37.8)	148 (37.6)	21 (5.3)	None
The expected overall benefits of cloud technology would outweigh its expected costs.	10 (2.5)	174 (44.2)	170 (43.1)	28 (7.1)	12 (3.0)	None
The return on investment from cloud technology would be unfavorable.⁎	2 (0.5)	63 (16.0)	183 (46.4)	133 (33.8)	13 (3.3)	None
The return on investment from cloud technology would be financially favorable.	7 (1.8)	185 (47.0)	160 (40.6)	33 (8.4)	9 (2.3)	Gender: females vs males agreeing/strongly agreeing, 52.8% vs 38.2%; *p* = 0.009; Cramér's V = 0.13
The startup cost of cloud computing is acceptable for adoption in pharmacy services.	8 (2.0)	152 (38.6)	206 (52.3)	20 (5.1)	8 (2.0)	None

Security and privacy						
Privacy of pharmacists' information is a major concern in adopting cloud technology.⁎	34 (8.6)	183 (46.4)	96 (24.4)	60 (15.2)	21 (5.3)	None
Adopting cloud computing would increase risks to patients' privacy.⁎	15 (3.8)	125 (31.7)	90 (22.8)	150 (38.1)	14 (3.6)	None
Using paper-based work instead of information technology is a more effective way to protect patient privacy.⁎	9 (2.3)	105 (26.6)	107 (27.2)	149 (37.8)	24 (6.1)	None
Using local in-house systems rather than cloud-based systems would better protect security and privacy.⁎	6 (1.5)	151 (38.3)	128 (32.5)	93 (23.6)	16 (4.1)	Gender: males vs females agreeing/strongly agreeing, 50.0% vs 35.9%; *p* = 0.010; Cramér's V = 0.13
Adopting cloud computing would improve information transfer compared with paper-based work.	73 (18.5)	229 (58.1)	67 (17.0)	12 (3.0)	13 (3.3)	Gender: females vs males agreeing/strongly agreeing, 80.3% vs 67.3%; *p* = 0.006; Cramér's V = 0.14
Accessing patient records anytime, anywhere, and from different devices would provide benefits that outweigh security and privacy risks.	35 (8.9)	204 (51.8)	97 (24.6)	51 (12.9)	7 (1.8)	None

Access, backup, and recovery						
Cloud computing could protect pharmacy data against loss caused by document loss, hardware failure, software failure, fire, or theft.	47 (11.9)	234 (59.4)	74 (18.8)	31 (7.9)	8 (2.0)	None
Internet speed and availability are major concerns for daily backup and recovery using cloud technology.⁎	47 (11.9)	252 (64.0)	63 (16.0)	22 (5.6)	10 (2.5)	None
The ability to work offline is a major benefit of using in-house systems.⁎	25 (6.3)	209 (53.0)	121 (30.7)	30 (7.6)	9 (2.3)	None
Limited hardware storage is a major concern when using in-house systems.	20 (5.1)	214 (54.3)	133 (33.8)	24 (6.1)	3 (0.8)	Gender: females vs males agreeing/strongly agreeing, 63.4% vs 49.1%; *p* = 0.010; Cramér's V = 0.13

Values in the response columns are presented as n (%), with percentages calculated from the total valid sample (*n* = 394). Percentages reported in the significant demographic association column are cross-tabulation percentages within the relevant demographic subgroup; for example, they represent the percentage of females versus the percentage of males, or pharmacists aged ≤35 versus >35 years, who agreed/strongly agreed with the item.

A Note on Theoretical Mapping: Questionnaire domains correspond to Rogers' Diffusion of Innovations (DOI) constructs as follows: perceived usefulness and service quality reflect relative advantage; security, privacy, and backup/recovery reflect compatibility; and cost evaluations reflect complexity. DOI theory was utilized as an interpretive framework to organize findings rather than as directly validated psychometric constructs.

Demographic variables examined were gender, age group, education level, place of work, and years of experience.

⁎ Negatively worded items are presented in their original wording; these items were reverse coded only for scale-level interpretation. Significant demographic associations are reported only when *p* < 0.05. Cramér's V indicates the strength of association.

**Table 4:** Predictors of readiness for cloud-service adoption (supportive, *n* = 280, vs. uncertain/non-supportive, *n* = 114).

Predictors	Sig, *P* value	Odds Ratio	95% Confidence Interval for Odds Ratio	
			Lower	Upper
Gender (Male Reference)	0.010	7.024	1.60	30.72
Age (>35 Reference)	0.000	5.778	2.31	14.39
Education level	0.83	1.968	0.26	17.33
Place of work Groups	0.06	0.479	0.22	1.03
Years of experience	0.19	5.110	0.44	58.81

### 4.2 Description and bivariate analysis related to statement of cloud using parameters

Overall, respondents showed a favorable orientation toward cloud-enabled pharmacy services. Most participants agreed or strongly agreed that cloud computing services could be useful for the pharmacy sector (89.1%), and an even higher proportion supported adopting more electronic services in pharmaceutical practice (93.7%). Similarly, high agreement was observed for the perceived ability of cloud technology to improve service quality (89.6%) and service flexibility (89.1%). These findings indicate strong perceived usefulness, positive digital orientation, and favorable expectations regarding the operational value of cloud-enabled pharmacy services. Among the demographic variables examined, only age was significantly associated with perceived quality improvement through cloud adoption, with pharmacists aged ≤35 years showing higher agreement than those >35 years (92.1% vs. 81.5%; *p* = 0.004; Cramér's V = 0.14), the effect size was moderate.

#### 4.2.1 Cost and value evaluation

Cost-related responses showed a more cautious pattern. Across several cost and value items, “not sure” responses ranged from 36.0% to 52.3%, indicating considerable uncertainty regarding the financial implications of cloud adoption. Only 32.0% of respondents agreed or strongly agreed that cloud technology would reduce the cost of pharmaceutical services, while 36.0% were uncertain. Similarly, 46.7% agreed or strongly agreed that the overall financial and non-financial benefits of cloud technology would outweigh its expected costs, whereas 43.1% were uncertain. Perceived return on investment also showed cautious optimism: 48.8% agreed or strongly agreed that cloud adoption would be financially favorable, but 40.6% remained unsure. These findings suggest that pharmacists may recognize the potential value of cloud services, but cost reduction, startup cost, and return on investment remain areas of uncertainty.

Gender was significantly associated with selected cost and value perceptions. Female pharmacists were more likely than male pharmacists to agree that cloud adoption could reduce pharmaceutical service costs (71.5% vs. 59.1%; *p* = 0.018; Cramér's V = 0.12). Female pharmacists were also more likely to perceive cloud systems as financially profitable (52.8% vs. 38.2%; *p* = 0.009; Cramér's V = 0.13). In contrast, male pharmacists showed stronger agreement that local in-house systems may provide better security and privacy than cloud-based systems (50.0% vs. 35.9%; *p* = 0.010; Cramér's V = 0.13). Female pharmacists showed higher agreement that cloud computing improves information transfer compared with paper-based work (80.3% vs. 67.3%; *p* = 0.006; Cramér's V = 0.14), and were also more likely to view limited hardware storage as a concern when using in-house systems (63.4% vs. 49.1%; *p* = 0.010; Cramér's V = 0.13).

#### 4.2.2 Security and privacy

Security and privacy responses showed a mixed pattern, indicating that perceived cloud benefits coexisted with continuing concerns about data protection. More than half of respondents agreed or strongly agreed that pharmacists' information privacy is a major issue in cloud adoption, while 35.5% agreed or strongly agreed that cloud computing may increase risks to patient privacy. At the same time, respondents did not clearly prefer paper-based systems as a privacy solution, as 43.9% disagreed or strongly disagreed that paperwork is more effective than information technology for protecting patient privacy. Similarly, 76.6% agreed or strongly agreed that cloud computing would improve information transfer compared with paper-based work, and 60.7% agreed or strongly agreed that anytime, anywhere, multi-device access to patient records may provide benefits that outweigh security and privacy risks. These findings suggest that pharmacists recognized the operational and communication advantages of cloud systems, but privacy and security concerns remain important implementation barriers.

Gender was significantly associated with two security/privacy items: male pharmacists were more likely than female pharmacists to agree that local in-house systems provide better security and privacy than cloud-based systems (50.0% vs. 35.9%; *p* = 0.010; Cramér's V = 0.13), whereas female pharmacists were more likely to agree that cloud computing improves information transfer compared with paper-based work (80.3% vs. 67.3%; *p* = 0.006; Cramér's V = 0.14). The effect sizes were small.

#### 4.2.3 Access, backup, and recovery

Backup and recovery items reflected a generally favorable perception of cloud-based data protection, but also highlighted infrastructure-related concerns. Most respondents agreed or strongly agreed that cloud computing could protect information in cases of data loss, document theft, hard-copy loss, hardware failure, software failure, or fire. Similarly, 59.4% agreed or strongly agreed that limited hardware storage is a major concern when using in-house systems, supporting the perceived value of cloud-based storage capacity. However, respondents also identified practical barriers to cloud-based backup and recovery, with 75.9% agreeing or strongly agreeing that internet speed and availability are major concerns for daily data backup and recovery. In addition, 59.3% agreed or strongly agreed that the ability to work offline is a major benefit of in-house technology. These findings suggest that pharmacists valued cloud systems for data protection and storage flexibility, but readiness may depend on reliable internet connectivity, offline-continuity options, and clear backup/recovery protocols.

Gender was significantly associated with perceived hardware-storage limitations, with female pharmacists more likely than male pharmacists to view limited in-house storage as a concern (63.4% vs. 49.1%; *p* = 0.010; Cramér's V = 0.13), again with a moderate effect size.

### 4.3 Multivariable analysis

The multivariable logistic regression model was statistically significant overall (Omnibus χ^2^ = 469.958, df = 9, *p* < 0.001, Nagelkerke R^2^ = 0.929), indicating that the included demographic and professional variables collectively predicted readiness for adopting cloud services (measured via support categories). The model showed acceptable calibration based on the Hosmer–Lemeshow goodness-of-fit test (χ^2^ = 11.159, df = 7, *p* = 0.132).

The receiver operating characteristic analysis showed that the multivariable logistic regression model had good discriminatory ability for identifying pharmacists classified ready (supportive, *n* = 280, 71.1%) versus not ready (uncertain/non-supportive, *n* = 114, 29.9%) regarding cloud-service adoption**.** The area under the ROC curve (Fig. 1) was 0.820 (SE = 0.070, *p* = 0.001; 95% CI: 0.683–0.956), indicating that the model distinguished reasonably well between ready/supportive pharmacists and those classified as uncertain or non-supportive. The dependent variable participants classified as ready/supportive (*n* = 280, 71.1%) and 114 classified as non-ready, uncertain, or non-supportive (28.9%).

**Fig. 1:**
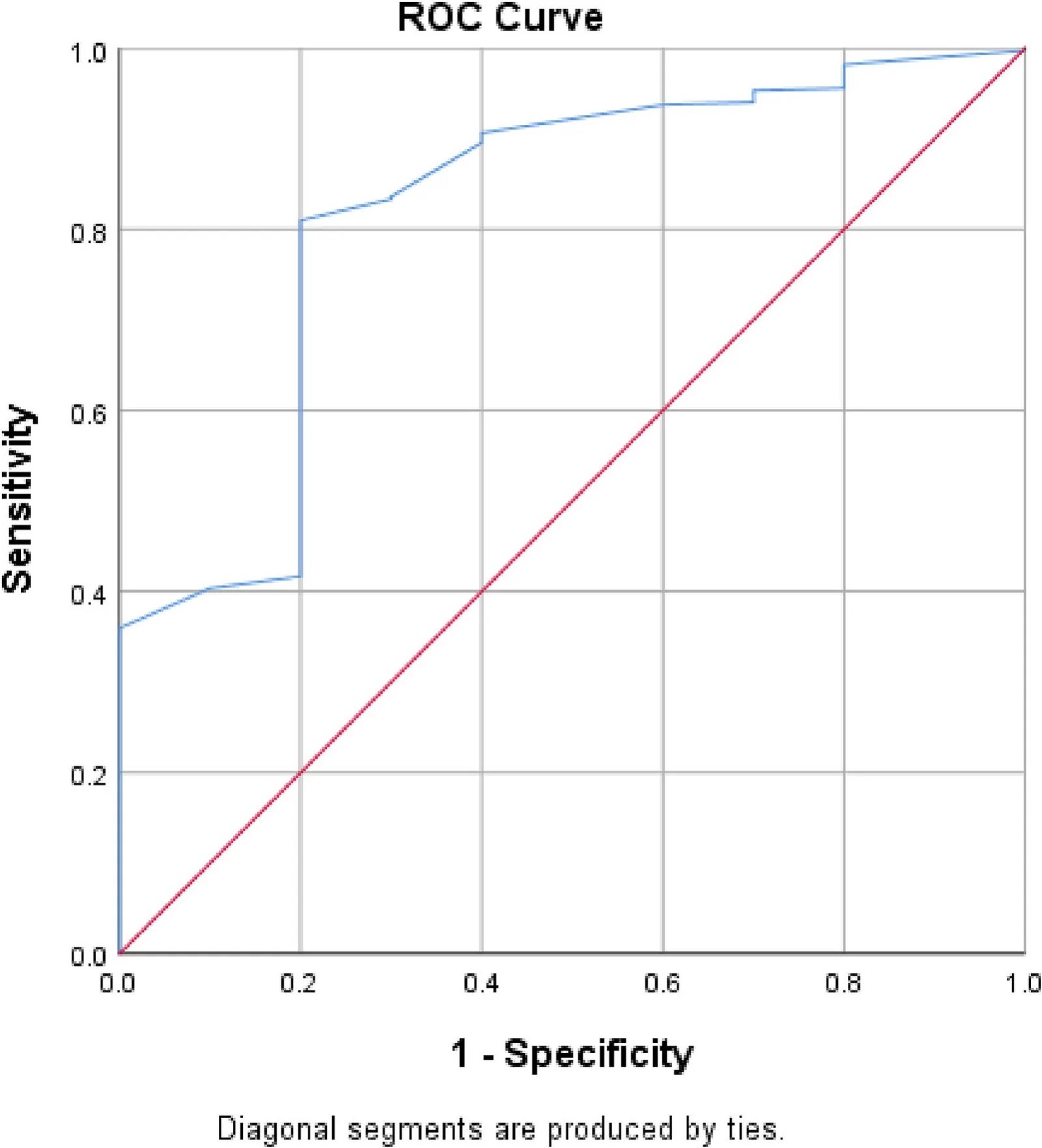
Receiver operating characteristic (ROC) curve for the multivariable logistic regression model predicting readiness for adopting cloud services. The area under the curve was 0.820 (95% CI: 0.683–0.956; *p* = 0.001).

Gender and age were significant predictors. Female pharmacists had higher odds of supporting cloud services than male pharmacists (OR = 7.024, 95% CI: 1.60–30.72; *p* = 0.010). Similarly, pharmacists aged ≤35 years had higher odds of supporting cloud services than those aged >35 years (OR = 5.778, 95% CI: 2.31–14.39; *p* < 0.001). See Table 4.

Education level, place of work, and years of experience were not statistically significant predictors of cloud-service support. Specifically, education level was not significantly associated with support (OR = 1.968, 95% CI: 0.26–17.33; *p* = 0.83), nor was place of work (OR = 0.479, 95% CI: 0.22–1.03; *p* = 0.06) or years of experience (OR = 5.110, 95% CI: 0.44–58.81; *p* = 0.19).

### 4.4 Proposed framework: cloud computing adoption readiness and decision process

Based on the quantitative findings, a proposed data-informed framework was developed to summarize the main factors shaping cloud computing adoption readiness in community pharmacy. The framework organizes the study findings (Fig. 2) where the main enablers were perceived usefulness, expected improvement in service quality, service flexibility, information transfer, backup/recovery, and cloud storage capacity. The main barriers or implementation concerns were cost, start-up uncertainty, internet reliability, offline continuity, pharmacists' and patients' privacy, and security concerns. These elements were derived from the response patterns observed across the attitude, cost-value, security/privacy, and backup/recovery domains.

**Fig. 2:**
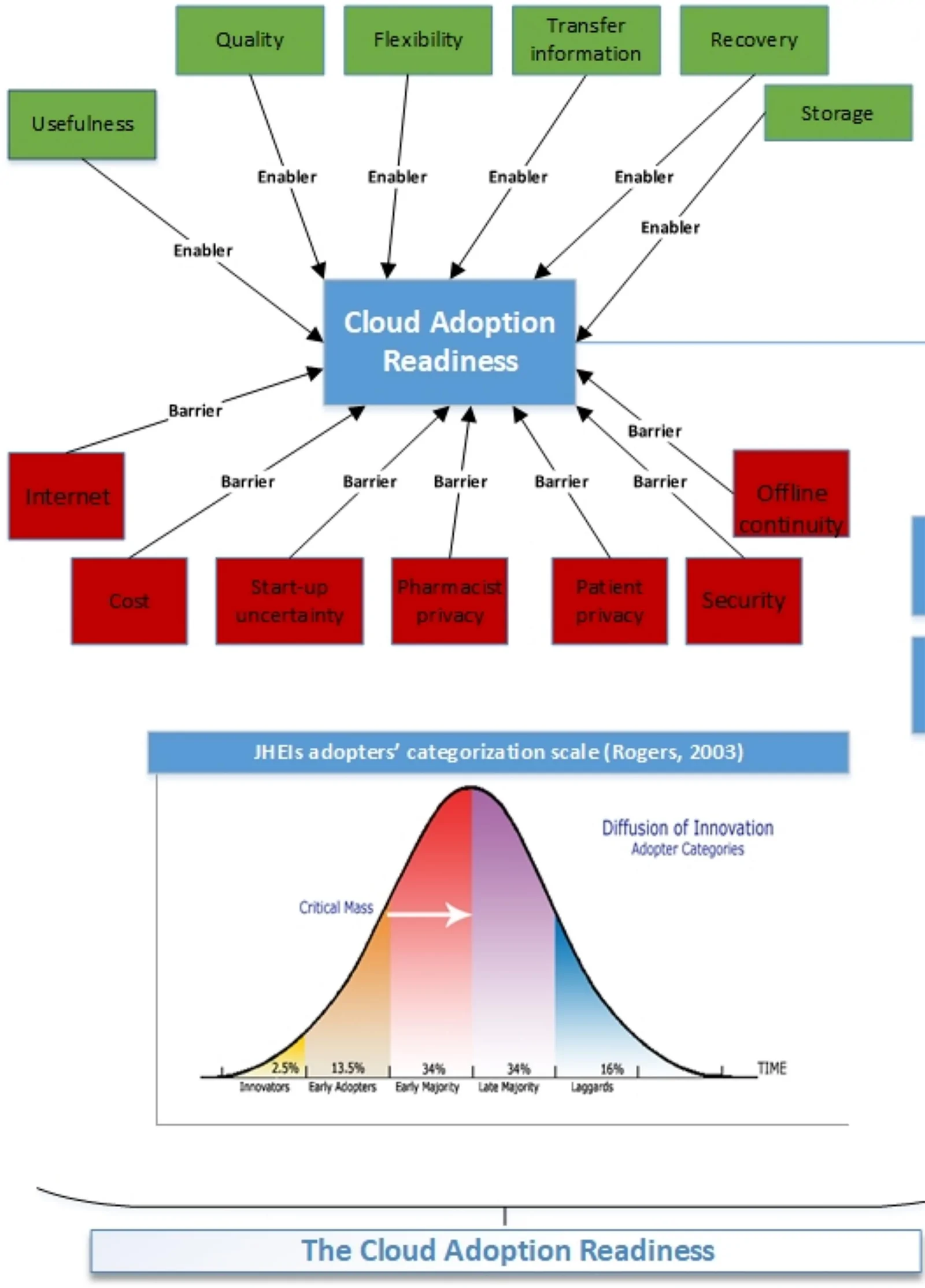
Framework for Cloud Computing Adoption Readiness in Community Pharmacy. The upper panel summarizes the study-derived enablers and implementation concerns influencing cloud adoption readiness. The lower panel presents a Diffusion of Innovations (DOI) adoption pathway, emphasizing how the early stages accurately reflect the baseline readiness and perspectives of early-adopting pharmacists.

The second component (Fig. 2) presents a theory-informed adoption pathway based on the DOI framework. This pathway illustrates how pharmacy cloud adoption progresses from initial knowledge and persuasion to decision-making, implementation, and confirmation. The inclusion of the DOI curve is specifically intended to contextualize the multivariable finding that pharmacists aged ≤35 years showed higher odds of supporting cloud services, reflecting how early-adopter characteristics naturally cluster within younger professional cohorts. While the framework models broader theoretical diffusion, its early stages align closely with the empirical readiness observed in this study, highlighting how initial uptake is driven by these foundational evaluation and persuasion phases.

## 5 Discussion

Pharmacists demonstrated strong receptiveness to cloud computing—driven by perceived utility, service flexibility, and backup capabilities—though tempered by practical concerns regarding costs, internet reliability, and data privacy. Multivariable analysis identified younger age and female gender as independent factors associated with cloud-adoption readiness (expressed as support), suggesting that this readiness state may be shaped more by digital orientation and perceived implementation confidence than by formal qualification, workplace type, or professional seniority. Given the cross-sectional design, these findings reflect statistical associations rather than causal determinants. Furthermore, when evaluating the magnitude of these associations—notably the wide confidence intervals observed for gender—these estimates should be interpreted with caution and require confirmation in future prospective and larger-scale studies.

The pattern of responses suggests that pharmacists' acceptance of cloud computing is anchored in its functional proximity to everyday pharmacy work, consistent with technology-acceptance evidence that users are more likely to adopt systems they perceive as useful for improving task performance.^45^, ^46^ Support was strongest for domains that pharmacists can immediately connect to service continuity, workflow efficiency, data access, and recovery from operational disruption. This is important because adoption is more likely when an innovation is perceived as offering a clear relative advantage over existing practice, rather than adding a separate technological burden.^9^, ^35^ Health-sector, particularly, community-pharmacy evidence supports this interpretation by showing that digital technologies can strengthen access to medication information, documentation workflows, interprofessional communication, and pharmacist decision-making, but their value depends on usability, interoperability, workflow fit, and pharmacy-specific implementation evaluation.^18^, ^21^, ^23^ Therefore, in the present study, pharmacists' support for cloud computing should be interpreted less as enthusiasm for technology itself and more as recognition of its potential to solve practical pharmacy-workflow problems.

The uncertainty around cost, return on investment, internet reliability, offline continuity, and privacy/security indicates an implementation-readiness gap, rather than simple resistance to cloud adoption. In technology-acceptance terms, perceived usefulness must be accompanied by facilitating conditions, risk control, and operational trust before users are likely to support sustained implementation.^46^ This is particularly relevant to community pharmacy, where digital tools must be usable, interoperable, workflow-compatible, and evaluated within pharmacy-specific practice conditions.^21^, ^23^ Evidence from medicines-optimisation research across care interfaces reinforces this interpretation, showing that poor interoperability, limited availability of digital data, and disruption to established workflows can reduce the practical value of digital tools.^33^ These concerns are closely aligned with the uncertainty observed in the present study regarding information transfer, connectivity, offline continuity, and routine service reliability. Accordingly, concerns related to connectivity, data protection, workflow continuity, and operational reliability should be addressed as core adoption conditions in community pharmacy services, not simply as secondary technical issues.^47^

Results from the multivariable analysis extend the community-pharmacy digitalisation literature by showing that support for cloud computing was independently associated with age and gender, but not with education level, workplace category, or years of experience. This contrast is important: whereas recent Jordanian community-pharmacy informatics research linked positive informatics attitudes with age, gender, education, and experience, the present adjusted model retained only age and gender, suggesting that cloud-service support may capture a more specific adoption signal than general digital receptiveness.^48^ This suggests that cloud-readiness may be shaped less by conventional professional markers and more by users' orientation toward technological change. The age association is theoretically coherent with Diffusion of Innovations theory, where earlier engagement with innovation is linked to perceived relative advantage, lower complexity, and greater tolerance of early implementation uncertainty; However, it should not be mapped directly onto Rogers' adopter categories: innovators represent the earliest 2.5% of adopters and early adopters the subsequent 13.5%, whereas this study more cautiously identifies younger pharmacists as a relatively adoption-ready subgroup.^35^ The gender finding adds a further signal for future pharmacy-specific research, but the wide confidence interval should be carefully considered.

### 5.1 Practical implications

The findings have direct implications for digital transformation planning in community pharmacy management. Cloud adoption in community pharmacy should not be introduced as a purely technical upgrade; it should be framed as a practice-change process linked to service quality, workflow flexibility, information transfer, backup and disaster recovery, and continuity of patient-service delivery. Directly addressing the empirical findings—where 75.9% of respondents identified internet reliability as a major concern and 59.3% valued offline functionality—implementation plans must incorporate concrete technological safeguards. Specifically, pharmacies should adopt hybrid architectures featuring robust offline functionality and clear downtime procedures to ensure continuous dispensing during connectivity failures. Furthermore, to address pharmacists' confidence in cloud data protection, vendors must provide automated backup redundancy and multi-tiered data-recovery mechanisms.

To resolve the widespread financial uncertainty observed in our survey (where 36.0% to 52.3% of pharmacists remained unsure about cost reduction and return on investment), technology providers must establish transparent vendor cost structures, avoiding hidden fees and offering predictable subscription models tied to measurable utility. Moreover, implementation should be evaluated not only through technical performance indicators, but also through pharmacy-practice outcomes, including medication-service continuity, documentation quality, workflow burden, patient-data access, and pharmacist confidence in routine use.

For pharmacy owners and technology vendors, the results support phased implementation through pilot testing rather than immediate full-scale replacement of existing systems. Hybrid or staged cloud models may be especially appropriate in settings where pharmacists value cloud storage, multi-device access, and backup capacity but remain concerned about internet dependence and offline service continuity. Vendor demonstrations should therefore move beyond general claims about digital efficiency and provide pharmacy-specific evidence on usability, interoperability, explicit cybersecurity safeguards (such as multi-factor authentication, role-based access control, and end-to-end encryption to mitigate privacy risks identified by respondents), downtime management, staff training, and operational costs.

For regulators, professional bodies, and academic institutions, the proposed framework can guide readiness assessment before wider cloud deployment. Targeted staff training programs must be instituted to bridge the adoption gap—particularly addressing demographic subgroup variations—focusing on both technical literacy and implementation confidence. Regulatory guidance should also clarify minimum expectations for patient-data protection, access control, audit trails, backup frequency, disaster recovery, and responsibilities of cloud-service providers. In this way, the study findings can inform practical adoption roadmaps that align technological readiness with professional trust, patient safety, and pharmacy-service continuity.

### 5.2 Limitations and future work recommendations

This study has several limitations. First, the cross-sectional design does not allow causal interpretation or assessment of whether stated readiness translates into actual adoption behavior after implementation. Second, the study measured pharmacists' perceptions and self-reported support for cloud-service adoption rather than observed use of a specific cloud-based pharmacy management system. Therefore, the findings should be interpreted as readiness signals, not as evidence of post-implementation effectiveness, patient-safety benefit, cost saving, or workflow improvement. Third, voluntary online recruitment may have overrepresented pharmacists who were more digitally connected or interested in cloud computing. Although multiple recruitment routes were utilized to broaden outreach, and anonymity and response screening supported response quality, these measures do not establish population representativeness. While this digital skew limits broad generalization to technology-averse sectors, the sample represents the essential ‘early adopter’ vanguard whose readiness and feedback guide initial cloud deployment. Selection bias may have influenced both the estimated level of support and the observed demographic associations, the magnitude of which could not be precisely determined. The findings should therefore be interpreted as perceptions and associations within the responding sample, with limited generalizability to the wider population of pharmacists. Fourth, the sample was predominantly female and aged ≤35 years, which reflects the respondent profile but may limit generalizability to older pharmacists, pharmacists in less digitally connected settings, and pharmacy owners with direct responsibility for technology investment decisions. Furthermore, while respondents comprised licensed responsible pharmacists, full-scale technology adoption also involves IT personnel and technical support. Consequently, the findings primarily reflect pharmacist perspectives on operational readiness, while comprehensive implementation requires broader stakeholder engagement. Fifth, the questionnaire was developed for the study and demonstrated content validity and satisfactory internal consistency, but further psychometric testing is needed to confirm its dimensional structure and predictive validity in other pharmacy populations. Finally, the proposed framework was developed as a data-informed and theory-informed synthesis of the present findings; it should not be interpreted as a validated prediction model.

Future research should employ stratified sampling across diverse practice environments to validate these insights within the broader pharmacy workforce, while future work should move beyond perception-based readiness assessments toward longitudinal and implementation-focused research. Prospective studies are needed to examine whether pharmacists' baseline readiness predicts actual adoption, sustained use, and post-implementation satisfaction after cloud-based pharmacy management systems are introduced. Pilot implementation studies should also evaluate measurable outcomes, including workflow efficiency, medication-safety indicators, service continuity during internet disruption, backup and recovery performance, user satisfaction, privacy and cybersecurity safeguards, and cost-effectiveness. In addition, future research should validate the proposed framework using stratified sampling across broader populations, compare independent and chain pharmacies, include technology decision-makers, and examine patient perspectives where patient-facing cloud services are involved.

## 6 Conclusion

This cross-sectional study shows that Jordanian pharmacists with community-pharmacy experience generally perceive cloud computing as useful for pharmacy practice and support the wider adoption of electronic pharmaceutical services. Strong support was observed for service quality, service flexibility, information transfer, backup and recovery, and reduced dependence on limited in-house hardware. However, this positive orientation was accompanied by important implementation concerns, particularly uncertainty about cost, return on investment, start-up cost, internet reliability, offline continuity, and the privacy and security of pharmacists' and patients' information.

In the adjusted analysis, younger age and female gender were independently associated with higher readiness for cloud-service adoption (supportive status), whereas education level, place of work, and years of experience were not statistically significant predictors. These findings suggest that readiness for cloud adoption may be influenced more by digital orientation and implementation confidence than by conventional professional characteristics alone. The proposed Cloud Computing Adoption Readiness framework provides a practical synthesis of the study-derived enablers, concerns, and decision pathway, but it requires further empirical validation before being used as a predictive or evaluative tool.

Overall, cloud computing adoption in community pharmacy should be treated as a structured practice-transformation process rather than a simple software transition. Successful implementation will require credible financial planning, reliable connectivity, offline-continuity options, transparent privacy and security safeguards, user training, and pharmacy-specific governance.

Future research should evaluate actual cloud-system implementation and determine whether readiness translates into measurable improvements in operational efficiency, service quality, medication safety, and patient-care continuity.

## Declaration of generative AI and AI-assisted technologies in the manuscript preparation process

During the preparation of this work, the authors used ChatGPT (OpenAI) as a language-support tool to assist with Arabic–English translation, editing, wording clarification, and improving the coherence and academic style of the manuscript. No AI tools were used for data collection, data analysis, or the generation or interpretation of study results. After using this tool, the authors thoroughly reviewed, revised, and edited all content and took full responsibility for the integrity, accuracy, originality, and final content of the manuscript.

## CRediT authorship contribution statement

**Mohanad Odeh:** Conceptualization, Methodology, Project administration, Supervision, Validation, Writing – review & editing. **Dana M. Odeh:** Formal analysis, Software, Validation, Writing – original draft. **Wafa Hourani:** Data curation, Investigation, Software, Visualization, Writing – original draft. **Ahmad Shadfan:** Data curation, Methodology, Software, Visualization, Writing – original draft. **Feras Jirjees:** Conceptualization, Investigation, Project administration, Supervision, Writing – review & editing.

## Funding

This research did not receive any specific grant from funding agencies in the public, commercial, or not-for-profit sectors.

## Declaration of competing interest

The authors declare that they have no known competing financial interests or personal relationships that could have appeared to influence the work reported in this paper.

## Data Availability

The data may be made available from the corresponding author upon reasonable request and subject to institutional approval.
